# Analysis of Mortality from Carcinomas Primary Localized in Region of Lip, Oral Cavity and Pharynx in Central Serbia, 1999–2015

**Published:** 2020-02

**Authors:** Milos M. STEPOVIC, Dalibor STAJIC, Marija SEKULIC, Zlata RAJKOVIC, Nela DJONOVIC

**Affiliations:** 1. Faculty of Medical Sciences, University of Kragujevac, Kragujevac, Serbia; 2. Department of Hygiene and Ecology, Faculty of Medical Sciences, University of Kragujevac, Kragujevac, Serbia; 3. Institute of Public Health, Kragujevac, Serbia

**Keywords:** Lip neoplasm, Pharyngeal neoplasm, Mouth neoplasm, Serbia

## Abstract

**Background:**

Lip, oral cavity, and pharyngeal cancers have been globally estimated to account for about 3.8% of all cancer cases and 3.6% of cancer deaths. Mortality of these cancers is generally higher in developing than in developed countries. Overall cancer mortality rate in Serbia is one of the highest in the world. The aim of this study was to determine the mortality rate trends and the most common localization of lip, oral and pharyngeal cancers in Serbia.

**Methods:**

The study was conducted in 2018 as descriptive epidemiological study and included years from 1999 to 2015. The differences in standardized mortality rates and number of deaths were analyzed with regard to age, gender, and tumor localization. Linear trend and regression were used to determine mortality rate trend.

**Results:**

There was statistically significant difference in the number of deaths between men and women in the ages of 40 and over (*P* < 0.01); male/female cancer mortality ratio was 4.56:1. Generally, the most common localization of this carcinoma was hypopharynx. There was no statistically significant increase of mortality rates from these cancers for both genders (males: y = 4.77 + 0.42x, *P* = 0.069 % change = +20.35; females: y = 1.03 + 0.01x, *P* = 0.40 % change = +4) during 17-year period.

**Conclusion:**

Promotion of healthy habits, life-styles and regular inspection of mouth by patients and health professionals should be better prioritized especially in developing countries where implementing and improving national health prevention programs are essential.

## Introduction

Non-communicable diseases are major health and development challenges of the twenty-first century. Lip, oral cavity, and pharyngeal cancers have been globally estimated to account for about 3.8% of all cancer cases and 3.6% of cancer deaths (1). Oral and pharyngeal cancer (grouped together) is the sixth most common cancer in the world (2–8). Almost half of all oral cancers reported in Europe and US are located in tongue (9). Histopathologically, oral carcinoma is often a squamous cell carcinoma (90%) developed from mucosal epithelium (10).

Lip cancers are highly frequent in Australia (due to solar radiation) and in central and eastern Europe (associated with tobacco smoking), while nasopharyngeal cancers are most common in northern Africa and eastern/southeast Asia (1). Oral cancer is significantly more common in developing countries than in developed countries. It accounts for less than 5% of all cancers in the United States, Western Europe and Australia, but it is the third most common type of cancer and a leading cause of mortality in South Asian countries such as Bangladesh, India, Pakistan and Sri Lanka (9, 11). However, high incidence of oral and pharyngeal cancers was also reported in some European countries such as France, Denmark, and Spain. Hungary was identified as the country with the highest rate of morbidity and mortality of these cancers in Europe. High regional differences in the incidence and mortality rates of lip, oral and pharyngeal cancers can be explained by differences in lifestyles and exposure to risk factors such as smoking, alcohol abuse, genetics, fungus, chronic irritations in mouth, nutrition habits, socio-demographic factors and ethnicity predisposition (12). Human papilloma virus (HPV) and Epstein-Barr virus were also identified as important risk factors (1, 13).

According to the newest GLOBOCAN reports, overall cancer age-standardized mortality rate in Serbia is the second-highest in Europe and one of the highest in the world (14). This can be explained by the fact that Serbia is a developing country with low socioeconomic status, inadequate healthcare (caused by obsolete diagnostic and therapeutic equipment and a lack of prevention programs), and high prevalence of risk factors such as smoking, alcohol abuse and bad nutritional habits. Besides, there is still a serious concern about potential health consequences of environmental pollution associated with the civil wars in Serbia (including NATO bombing in 1999).

The aim of this study was to investigate the mortality rate trends of lip, oral and pharyngeal cancers in Serbia, as well as to determine the most common localization of these cancers. The study covered a 17-year period (1999–2015) that had not been included in previous studies conducted in Serbia. Besides, the mortality was investigated with regard to age and gender differences. Fifteen types of mouth and pharynx cancers were considered.

## Materials and Methods

This descriptive epidemiologic study was conducted in 2018 and used data on mortality rates and number of deaths from primary cancers localized in the lip, oral cavity and pharynx region. The data were obtained from fully published reports on the incidence and mortality of cancers from 1999 to 2015, provided by population Cancer Registry in Central Serbia “Dr Milan Jovanovic - Batut”. Reported cases of mortality from malignant tumors were registered only on the basis of death certificates. The Institute of Public Health in Kragujevac has permission for using the data. In accordance with the local legislation and institutional requirements, ethical review and approval were not required for this study. The deaths from carcinomas which were not primary localized in the region of lip, oral cavity and pharynx (metastatic carcinomas from other regions) were excluded.

Region of central Serbia includes following districts: Kolubara, Mačva, Raška, Moravica, Zlatibor, Rasina, Šumadija, Pomoravlje, Braničevo, Podunavlje, Zaječar, Bor, Nišava, Pčinja, Jablanica, Toplica, Pirot and the city of Belgrade. The age standardized rates (per 100,000 people per year) were calculated by direct standardization, using the World Standard Population (15). Malignant tumors of the mouth and throat region were encoded according to the Tenth International Classification of Disease (C00-C14). Death cases were classified into six age-categories.

Data were processed by Statistical Package for the Social Sciences software (SPSS Inc, version 21.0, Chicago, IL, US). Normality was assessed by Kolmogorov-Smirnov test. Mann Whitney and Kruskal-Wallis tests were used to compare the mortality between genders and among different age groups; results were significant at *P*<0.05. Linear trend and regression were used to determine mortality rate trend. Coefficient of determination from linear trend model was used to determine percentage of periodical changes of mortality while coefficient b was used to assess average annual mortality change.

## Results

During the 17-year observation period (1999–2015), 5094 people died in central Serbia from malignant tumors localized primary in lip, oral cavity and pharynx region. Of this total number of deaths, 3945 (77.4%) occurred in males and 1149 (22.6%) in females (Table 1).

**Table 1:** The number of death cases by age groups, and total number of death cases for males, females and both genders, and for ally localizations (1999–2015)

***Primary localization of carcinomas***	***Age groups(yr)***						***Σ M***	***Σ F***	***Σ M+ F***
	to 29	30–39	40–49	50–59	60–69	≥70			
Lip Base of tongue	1	1	4	30	64	243	240	103	343
	1	7	27	118	113	86	294	58	352
Other and unspecified parts of tongue	4	11	61	199	191	161	504	124	628
Gum Floor of mouth	0	0	4	11	10	13	26	12	38
	0	5	42	139	119	96	331	71	402
Palate Other and unspecified parts of mouth	1	1	21	45	51	47	120	46	166
	1	2	18	43	56	90	150	60	210
Parotid gland Other and unspecified major salivary glands	2	0	15	57	104	205	245	138	383
	0	4	9	21	24	69	71	56	127
Tonsil	1	2	24	103	99	104	266	67	333
Oropharynx	2	2	31	110	94	94	264	69	333
Nasopharynx	8	14	25	89	87	96	228	91	319
Pyriform sinus	0	0	0	3	5	5	11	2	13
Hypopharynx	7	7	118	334	378	241	905	181	1086
Other and ill-defined sites in lip, oral cavity and pharynx	3	5	38	97	110	107	290	71	361
Total	31	61	437	1399	1505	1657	3945	1149	5094

**Fig. 1:**
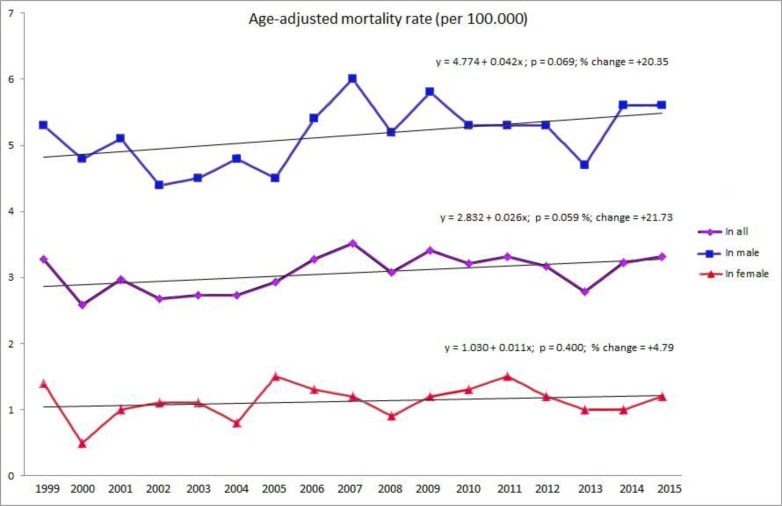
The mortality rates shown by gender and through years (1999–2015) from carcinomas primary localized in lip, oral cavity and pharynx region

Total standardized mortality rate was 3.07/100.000 (5.15/100.000 for males and 1.13/100.000 for females). The male/female cancer mortality ratio was 4.56:1. Standardized mortality rate trends were analyzed for males and females in the period from 1999 to 2015. There was no statistically significant difference in the mortality rates in the observed period (for all: y = 2.832 + 0.026x; *P* = 0.059 %; change = +21.73; for males: y = 4.77 + 0.42x; *P* = 0.069; % change = +20.35; for females: y = 1.03 + 0.01x; *P* = 0.40; % change = +4) (Fig. 1). Average annual change of mortality rate was + 0.026 (+0.42 for males and +0.01 for females). Linear trend model predicted that those rates would rise to 3.65/100.00 (6.08/100.000 for males and 1.37/100.000 for females) until 2030.

**Fig. 2:**
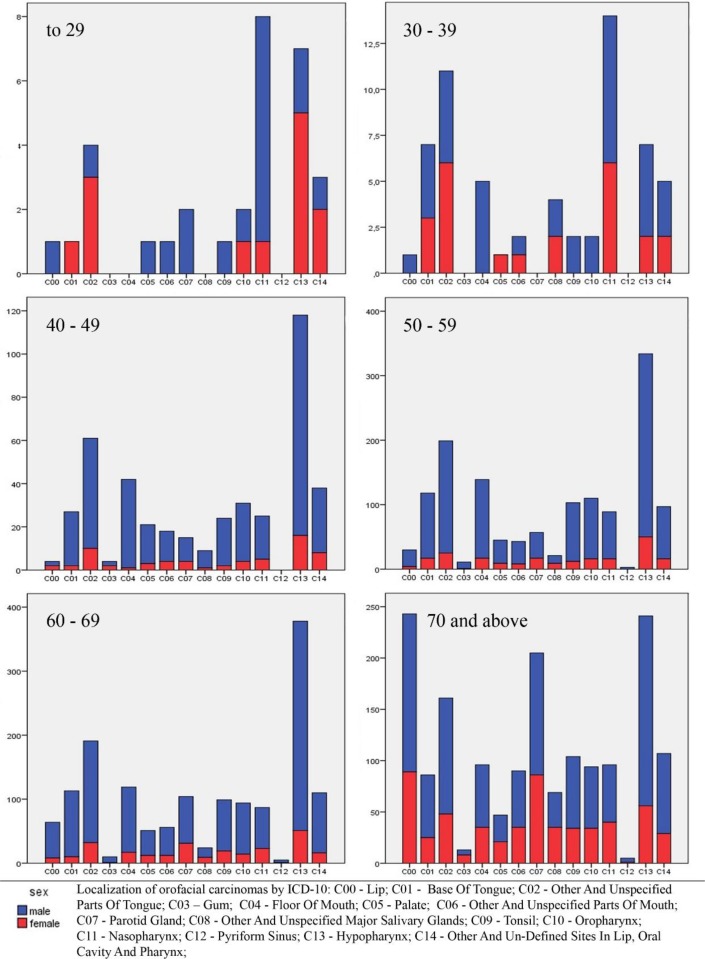
Number of deaths (in the period 1999–2015) from different types of cancer, with regard to age and gender

**Fig. 3:**
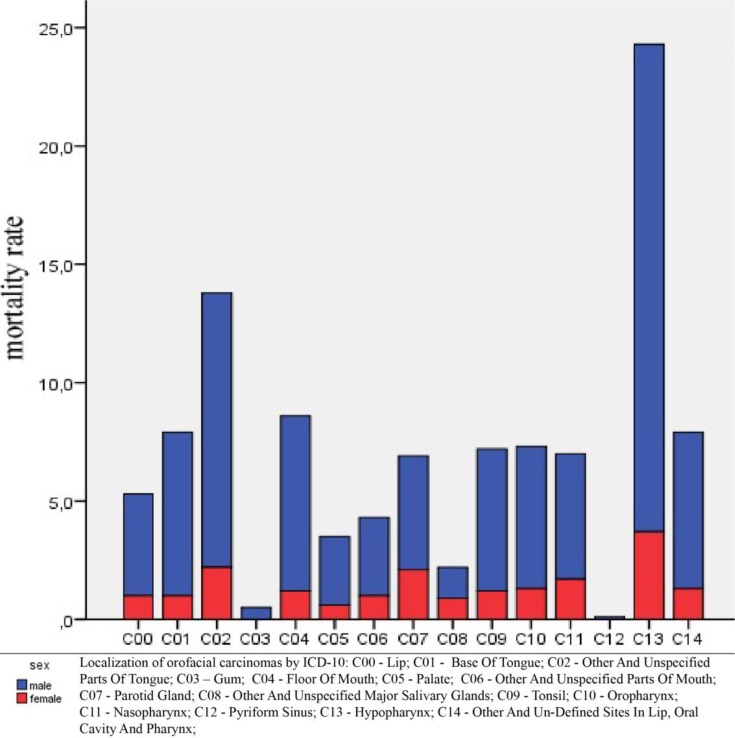
Mortality rates from different types of cancers in males and females

The number of deaths in males and females was analyzed with regard to age. The results show that there is a statistically significant difference in the number of deaths between males and females in the age group of 40–49 and above (*P* = 0.000), wherein the cancers are more common in males. When considering primary localization of cancers, there are statistically significant differences in all age groups (*P* = 0.000). For age groups from 40 to 70 (in both genders), dominant regions of localization were hypopharynx and other parts of the tongue. In the groups aged 70 and over, the most common localizations were hypopharynx, lips, and parotid glands (Fig. 2), while cancer of nasopharynx was the most common cause of death in the young age groups (< 40 y). Considering the average standardized mortality rates, regardless of age, it is possible to distinguish three dominant regions: hypopharynx, other parts of the tongue and floor of the mouth (Fig. 3).

## Discussion

Worldwide, there are big differences in the incidence and mortality rates of lip, oral cavity and pharynx carcinomas. According to the newest data provided by GLOBOCAN, age-standardized mortality rate of lip, oral and pharyngeal cancers in Serbia in 2018 was 3.8 (6.8 for males and 1.2 for females) per 100.000 (14). This report is in agreement with our results based on the 17-year period estimates: 3.07/100.000 (5.15/100.000 for males and 1.13/100.000 for females). The results of our study are also comparable to the values reported by Ilic et al. (6.2 and 1.2 per 100.000 for males and females, respectively) who investigated the mortality of lip, oral, and pharyngeal cancer in Serbia during the period 1991–2009 (16). When compared to the surrounding (developing) countries, Serbia has lower age-standardized mortality rate than Romania (6.1) and Hungary (8.2), but higher than its southern neighbors such as Albania (1.5), Montenegro (0.84) and the former Yugoslav Republic of Macedonia (1.3) (14). It is interesting to notice that Serbia borders directly with Hungary which has the highest mortality rate of lip, oral and pharyngeal cancers in the world (13,14). Such high regional differences in mortality rates are probably associated with different lifestyles and habits that have been identified as major risk factors such as alcohol use, tobacco smoking, unhealthy diet, human papilloma virus infection, etc.

The mortality rate of lip, oral and pharyngeal cancers increases with age; the highest number of cases in our study belonged to in the group aged 70 and over. This result is in agreement with previous reports (16). A slightly increasing trend in mortality rate was also observed for both genders. However, this trend was not statistically significant (*P*>0.05). Similar results were also reported by some other authors (16–18). Mihajlovic et al. (19) have predicted an increase of incidence and mortality rates of all leading cancers in Serbia. However, our results and predictions are far below the values predicted by Ferlay et al. for 2018 (20).

Our study has identified hypopharynx as the most common region of localization i.e. malignant tumors of this region made the largest contribution to the mortality rate. Previous studies have also shown that hypopharynx cancer has the worst prognosis of all head and neck squamous cell cancers. Such poor prognosis is due to the fact that hypopharynx has a rich submucosal lymphatic network that promotes early dissemination (21,22). Besides, hypopharynx lies below the oropharynx, and it is visually inaccessible by routine examination. Therefore, carcinomas of this region are often diagnosed and treated at a late-stage of disease.

According to Ilic et al. (16), the mortality rate of lip, oral cavity and pharynx cancers in Serbia (in the period 1991–2009) was 5.5 times higher in males than in females. Male to female mortality rate ratio obtained in our study (4.56) was slightly lower than the ratio reported by Ilic et al., but it fell within the range of 3–10 reported by Yako-Suketomo and Matsuda (23) for 11 countries in Asia, USA and Europe. Clearly, gender discrimination was observed in all studies. It can be explained by different lifestyles between genders. Serbia is a developing country where smoking and alcohol abuse are rather common. There are evidences that tobacco in various forms has carcinogenic effects in the oral cavity (24). Based on the National health survey conducted in Serbia in 2013, smoking prevalence in the population aged 15 and over was 34.7% and it was significantly higher in males than in females (25). Besides, studies suggest that the alcohol can be a major risk factor for oral cancers, particularly when combined with tobacco usage (26). Data from National survey showed that daily alcohol use was seven times higher in males than in females (25). Dietary habits have also been proposed as an important factor in cancer development. Previous studies have reported a lower risk of lip, oral cavity and pharynx cancer with higher intake of fruits and vegetables (27). More than half of the population in Serbia rarely or never consumes fruits and vegetables, but females use it more often than males (25). Previous studies have also pointed out the importance of other risk factors for oral neoplasms like HPV (particularly type 16), HSV-type 1, candidiasis, poor oral hygiene and prolonged irritations in mouth (28–31). The awareness of these risk factors is generally poor in the countries with low income, low level of education and inadequate level of health literacy.

In spite of numerous studies investigating the etiology of lip, oral and pharyngeal cancers, the prevalence of these diseases is still high. Preventive measures should be implemented in order to reduce the risk factors and increase the level of health literacy of the population. All oral ulcerations and wounds that do not heal properly during the period longer than 15 days should be examined by health professionals. Healthy lifestyles and eating habits should be an important part of patient education. Besides, screening for potentially carcinogenic viruses should be considered as a preventive routine. The promotion of healthy habits and regular inspection of the mouth region every six months should be prioritized better. These preventive programs should be included in national prevention programs.

This study provides useful information on mortality rate trends, the most common localizations and gender differences regarding lip, oral cavity and pharynx cancers in Serbia. It promotes implementation of preventive measures with the aim of early detection and treatment of these cancers. The data reported here may also prove useful for other developing countries with similar lifestyles, habits, socioeconomic status, and health system performances. However, the study was limited by the fact that the results were based on death certificates only, therefore included no data on potential risk factors and their impact on the incidence and mortality rates of lip, oral cavity and pharynx carcinomas.

## Conclusion

Mortality rates from carcinomas localized in the region of lip, oral cavity and pharynx are growing, not only in developing countries like Serbia, but in developed countries worldwide. Mainly due to the lifestyle, male gender is more likely to be affected, but recent years show a equalization in these odds, as the female rights became more equal. Reinforcing health systems and improving the preventive measures are the main keys in containing this public health problem and also conducting similar epidemiological studies and studies on potential causally consequential risk factors.
